# Supplementary material for: Evaluation of resistance to powdery mildew and identification of resistance genes in wheat cultivars
Source: PeerJ. 2021 Jan 11;9:e10425. doi: 10.7717/peerj.10425 (PMC7808266; doi:10.7717/peerj.10425)
Supplement: Supplemental Information 2 [file peerj-09-10425-s002.doc]

The raw data of Fig 1

| Isolate | The number of different ITs | | | | | |
| --- | --- | --- | --- | --- | --- | --- |
|  | 0 | 0; | 1 | 2 | 3 | 4 |
| 09558-1 | 3 | 1 | 1 | 3 | 18 | 43 |
| W1 | 8 | 4 | 2 | 9 | 22 | 24 |
| W12 | 7 | 2 | 4 | 2 | 18 | 36 |
| L14 | 5 | 3 | 3 | 4 | 24 | 30 |
| T7 | 3 | 4 | 5 | 1 | 27 | 29 |
| H1-5-1 | 3 | 1 | 3 | 1 | 20 | 41 |
